# Distinct prophage gene profiles of *Staphylococcus aureus* strains from atopic dermatitis patients and healthy individuals

**DOI:** 10.1128/spectrum.00915-24

**Published:** 2024-07-16

**Authors:** Zhongjie Wang, Xue Peng, Claudia Hülpüsch, Mohammadali Khan Mirzaei, Matthias Reiger, Claudia Traidl-Hoffmann, Li Deng, Michael Schloter

**Affiliations:** 1 Research Unit for Comparative Microbiome Analysis, Helmholtz Munich, German Research Center for Environmental Health, Neuherberg, Germany; 2 Faculty of Biology, Biocenter, Ludwig Maximilian University of Munich, Munich, Germany; 3 Institute of Virology, Helmholtz Munich, German Research Centre for Environmental Health, Neuherberg, Germany; 4 Environmental Medicine, Faculty of Medicine, University of Augsburg, Augsburg, Germany; 5 Insitute of Environmental Medicine, Helmholtz Munich, German Research Center for Environmental Health, Neuherberg, Germany; 6 Christine Kühne Center for Allergy Research and Education, Davos, Switzerland; 7 Chair of Prevention of Microbial Infectious Diseases, Central Institute of Disease Prevention and School of Life Sciences, Technical University of Munich, Freising, Germany; 8 Chair of Environmental Microbiology, TUM School of Life Sciences Weihenstephan, Technical University of Munich, Freising, Germany; University of West London, London, United Kingdom

**Keywords:** *Staphylococcus aureus*, atopic dermatitis, random forest, prophage, pathogenesis

## Abstract

*Staphylococcus aureus* strains exhibit varying associations with atopic dermatitis (AD), but the genetic determinants underpinning the pathogenicity are yet to be fully characterized. To reveal the genetic differences between *S. aureus* strains from AD patients and healthy individuals (HE), we developed and employed a random forest classifier to identify potential marker genes responsible for their phenotypic variations. The classifier was able to effectively distinguish strains from AD and HE. We also uncovered strong links between certain marker genes and phage functionalities, with phage holin emerging as the most pivotal differentiating factor. Further examination of *S. aureus* gene content highlighted the genetic diversity and functional implications of prophages in driving differentiation between strains from AD and HE. The HE group exhibited greater gene content diversity, largely influenced by their prophages. While strains from both AD and HE universally housed prophages, those in the HE group were distinctively higher at the strain level. Moreover, although prophages in the HE group exhibited variously higher enrichment of differential functions, the AD group displayed a notable enrichment of virulence factors within their prophages, underscoring the important contribution of prophages to the pathogenesis of AD-associated strains. Overall, prophages significantly shape the genetic and functional profiles of *S. aureus* strains, shedding light on their pathogenic potential and elucidating the mechanisms behind the phenotypic variations in AD and HE environments.

**IMPORTANCE:**

Through a nuanced exploration of *Staphylococcus aureus* strains obtained from atopic dermatitis (AD) patients and healthy controls (HE), our research unveils pivotal genetic determinants influencing their pathogenic associations. Utilizing a random forest classifier, we illuminate distinct marker genes, with phage holin emerging as a critical differential factor, revealing the profound impact of prophages on genetic and pathogenic profiles. HE strains exhibited a diverse gene content, notably shaped by unique, heightened prophages. Conversely, AD strains emphasized a pronounced enrichment of virulence factors within prophages, signifying their key role in AD pathogenesis. This work crucially highlights prophages as central architects of the genetic and functional attributes of *S. aureus* strains, providing vital insights into pathogenic mechanisms and phenotypic variations, thereby paving the way for targeted AD therapeutic approaches and management strategies by demystifying specific genetic and pathogenic mechanisms.

## INTRODUCTION

Atopic dermatitis (AD) is a chronic, recurrent, and inflammatory skin disease that affects up to 20% of children and 10% of adults worldwide, with an increasing prevalence in developed countries (1, 2). It is often associated with other health risks such as asthma and allergies (3). While the exact etiology of AD remains unknown, it is generally accepted that a complex interplay between genetics, immunology, and skin microbiome is involved in disease development and progression (4). Overall, AD is characterized by a dysbiosis of the skin microbiome with an overall reduced diversity (5). Specifically, the opportunistic pathogen *Staphylococcus aureus* is increased in both relative and absolute abundance on the skin of AD patients compared to healthy individuals (HE) (6–8), highlighting a significant association between *S. aureus* and AD. However, *S. aureus* also asymptomatically colonizes up to 30% of the human population (9). These findings suggest that substantial strain diversity exists within *S. aureus*, potentially explaining the variance between pathogenic and non-pathogenic strains (10, 11). This hypothesis has been proven in a recent study, where *S. aureus* strains from patients with severe and mild AD induce varying levels of inflammation in mouse models, with the degree of inflammation correlating directly to the severity of the disease of the patients where the strains were isolated from (12). In addition, AD patients are often characterized by a subject-specific clade or sequence type (ST) of *S. aureus* during disease development (12, 13). However, a recent pan-genome analysis revealed that *S. aureus* strains cannot be differentiated based on whole genome in terms of the health status of the subject the strains were isolated (14).

The genetic variability in bacterial populations is driven by mutation rates and horizontal gene transfer (HGT), the latter of which is predominantly facilitated by mobile genetic elements, which are mostly converted by conjugation or transduction (15). It has been suggested that the primary mechanism of HGT in *S. aureus* is transduction mediated by bacteriophages (16). Given their host specificity and ability to carry a diverse array of functional genes (17), bacteriophages can profoundly influence the evolution of their bacterial hosts. The genetic variation induced mainly by prophages, bacteriophages which integrated into bacterial genomes in a stable manner, not only enhances bacterial abilities to adapt to broader conditions but may also increase their pathogenicity with significant clinical implications (18). Despite the growing insights into the genetic diversity of *S. aureus* strains, there remains a significant gap in our understanding of their prophages and potential links to AD. Therefore, efforts to profile these prophages are essential for exploring the intricate relationship between genomic composition, phage-driven adaptation, and the resulting phenotypic outcomes.

Machine learning algorithms have been proposed as powerful and informative tools in phenotyping and microbial feature classification (19, 20). To address the knowledge gap in this study, we developed a random forest (RF) classifier for the accurate identification of marker genes differentiating AD-related *S. aureus* strains from those in HE. This classifier was employed using genomes from 300 strains obtained from public databases (AD: 150 vs HE: 150), complemented by 48 strains isolated and sequenced in our lab (AD: 33 vs HE: 15). Our methodology successfully identified a suite of marker genes, including the distinct prophage profiles, capable of distinguishing AD-associated strains with high accuracy. This study may not only highlight the significant importance of prophages on the gene content of *S. aureus*, as well as their role in the pathogenesis of AD, but might also stimulate the development of diagnostic markers to differentiate commensal strains and those with pathogenic potential.

## RESULTS

### RF classifier reliably identifies AD-related *S. aureus* strains

For our study, genomes of 348 *S*. *aureus* strains were used, including 183 from AD patients and 165 from HE. These *S. aureus* strains exhibited high completeness (>98%) and low contamination (<4%) (more details in Table S1). To identify genes that lead to genotypic variations, we developed an RF classifier based on the gene presence-absence table of *S. aureus* strains. Performance metrics showed constantly improving accuracy from 86.93% in the 5:5 training-test set to 91.67% in the 9:1 set and area under the curve (AUC) from 94.62% to 97.69% (Fig. S1). We selected the 9:1 partition due to its superior performance, yielding 90% accuracy and 94.67% AUC for the test data set (Fig. S2). After *k*-fold cross-validation (Fig. S3), we optimized the classifier by focusing on the 50 most informative “feature” genes. The optimized classifier achieved an accuracy of 90% and an AUC of 100% for the test data set (Fig. S2 and S4). On the real-world data set of 48 strains, the initial classifier attained 87.5% accuracy and 76.36% AUC, while the optimized classifiers reached 93.33% accuracy and 81.82% AUC (Fig. S2 and S4).

### Distribution and function of the “feature” genes in *S. aureus*

**Fig 1:**
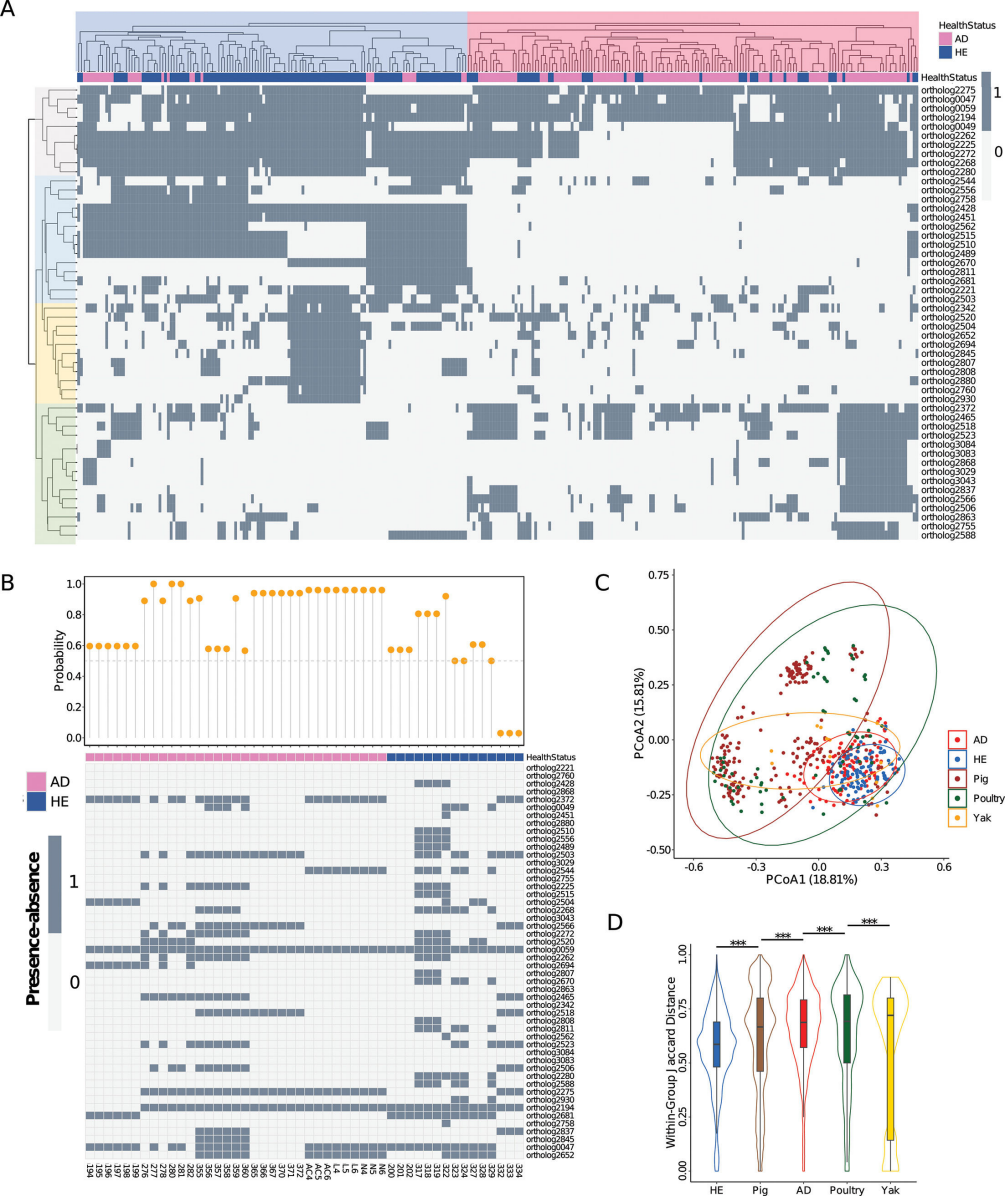
Distribution of the 50 marker genes in *S. aureus* strains from human and other hosts. (**A**) Heatmap of the 50 marker genes in the public data set. Dark gray indicates presence, and light gray indicates absence of marker genes. The strains (columns) form two main clusters shadowed in blue (HE cluster) and pink (AD cluster), respectively. Colors for health status are the same in B. The marker genes (rows) form four clusters. Both rows and columns were clustered using the Euclidean method. (**B**) Heatmap of the presence-absence of the 50 marker genes of the real-world data set and the prediction probability of the optimized classifier for each strain. The 50 marker genes (rows) are ranked by importance. (**C**) Principal coordinates analysis (PCoA) based on the marker genes of *S. aureus* strains from different hosts. Strains from pig (dark red), poultry (dark green), and yak (yellow), along with the strains from AD patients (red) and healthy controls (blue), are included for comparison. The ellipses denote the 95% confidence interval. Variances explained in the directions are shown in the parenthesis. (**D**) Within-group distance of the five different hosts. The Jaccard method was used for the calculation. The hosts are ranked by the medians in ascending order. The significance comparison of different hosts was performed using the Wilcoxon rank sum test. The significance of differences is shown between all pairs of groups, indicated by *P*-values <0.001 (***).

Based on our analysis of the distribution of feature genes, *S. aureus* strains formed two clusters using the genomes obtained from the 300 isolates (Fig. 1A). One cluster predominantly consisted of strains from HE (77.7%, HE cluster), while the other cluster was predominantly based on strains from AD (73.9%, AD cluster). In addition, four distinctive co-occurrence patterns emerged among the 50 feature genes (Fig. 1A); two groups were predominantly found in the HE cluster, whereas one group was more prevalent in the AD cluster, and the last group showed varied prevalence among its within-group genes. For the real-world data set, all strains from AD patients were accurately classified (Fig. 1B), with most prediction probabilities for each strain of the AD group surpassing 90%. The strains from HE had varying prediction probabilities between 50% and 90% despite three instances of misclassification. However, the absence of 10 feature genes, in particular the top 2 (phage holin and hypothetical protein), might explain the relatively lower prediction probabilities for some strains in this data set.

Since *S. aureus* possesses diverse STs, we also explored whether feature genes correlate more with health status (AD vs HE) or clonal structure (STs) due to asymmetrical ST sampling between AD and HE. ST analysis revealed that 78% of our strains exhibit matching STs, indicating that those with asymmetrical STs represent a relatively small subset. Furthermore, the identification of biomarkers with matched ST samples from AD and HE revealed that 31 biomarkers, accounting for 62%, remained consistent with the original set (Table S1). Notably, the top 22 biomarkers from this repeated analysis overlapped with those identified in the original study, contributing to an 84% prediction accuracy for these biomarkers. This classifier (Fig. S5) also demonstrated highly similar performance with the original version (Fig. S2). Moreover, the clustering analysis (Fig. S6) based on the presence of feature genes revealed a balanced distribution of major STs (e.g., ST1, 5, 8, 15) across strain clusters, and the phylogenetic analysis (Fig. S7) illustrated only a small proportion of strains exhibiting near-clonal similarity based on phylogenetic distance, further supporting our findings. Taken together, these findings suggest that the identified biomarkers are more significantly correlated with the health status of individuals rather than the asymmetrical distribution of STs, although the latter does have influence on the results to some extent.

To further investigate whether these genes can be used to discriminate *S. aureus* strains based on their hosts of origin, we did principal coordinates analysis (PCoA) including *S. aureus* strains isolated from pigs, poultry, and yaks (metadata of non-human isolates in Table S2). The PCoA result showed human-derived strains (both AD and HE groups) clustered separately (Fig. 1C). This suggests the ability of the “feature” genes to distinguish strains based on host origin. Additionally, significant within-group distance differences among hosts (Wilcoxon rank sum test, all *P*-values <0.001, Fig. 1D) emphasize the role of these genes in host-specific adaptations.

**Fig 2:**
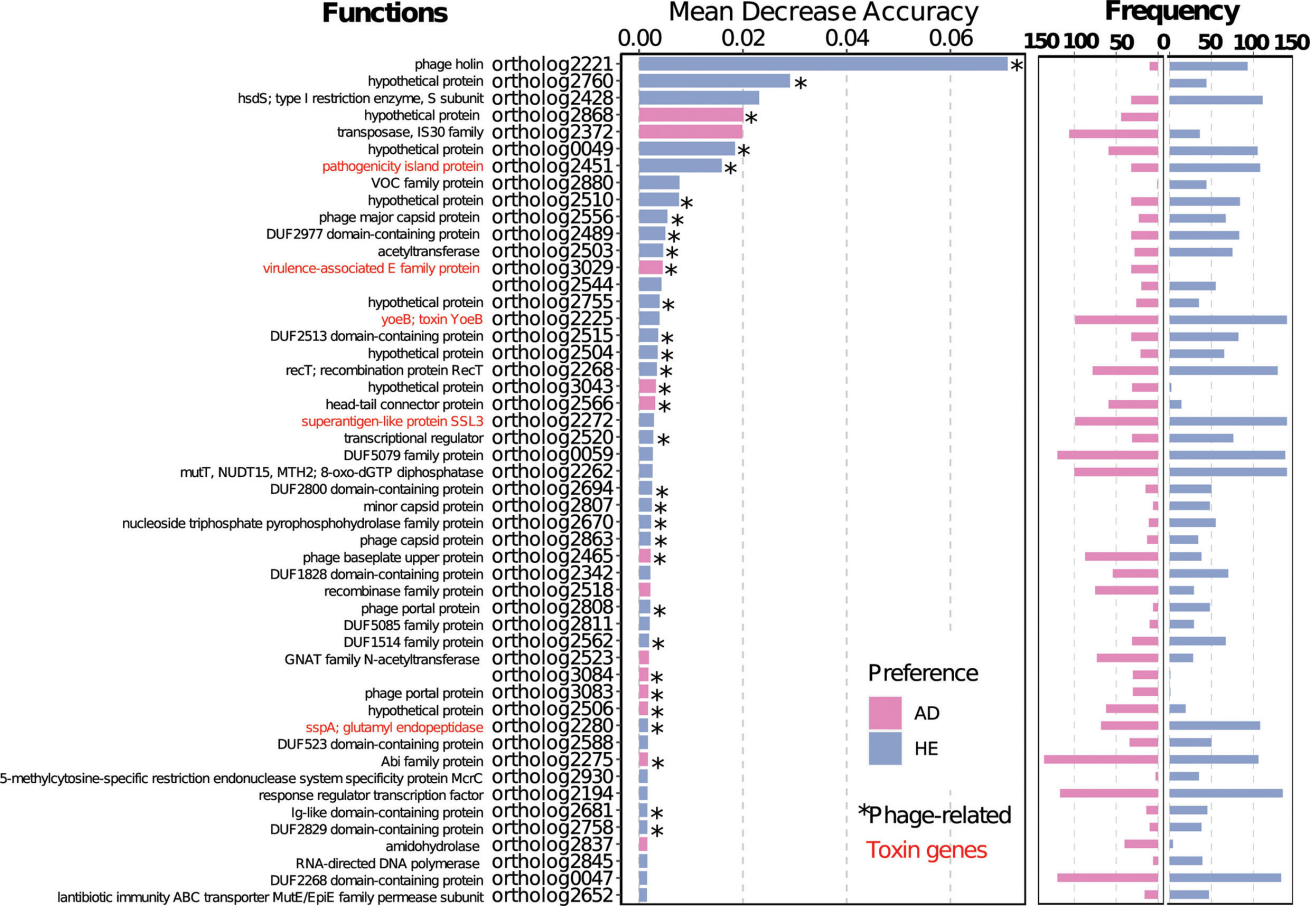
Function and frequencies of the most discriminative 50 marker genes as assigned by random forest classifier. Orthologs were assigned using MCL, and functions annotated using KEGG and Refseq. Toxin genes are labeled in red, assigned using the virulence factor database. Middle panel shows marker genes ranked by mean decrease in accuracy, as determined by an RF classifier. Bar color indicates the prevalence of genes in all AD (pink) or HE (light blue) strains. Genes marked with asterisks (*) are phage related, as assigned by blasting against predicted prophage genes. The right panel shows gene frequency in all AD and HE strains, respectively.

We subsequently examined the importance and functions of the “feature genes.” As shown in Fig. 2, a significant proportion of the genes, 37 out of the 50 (74%), were more commonly found in strains from HE, suggesting that the differentiation was predominantly influenced by gene families exclusive or more abundant in the HE group. Upon functional characterization of the genes, 32 genes (64%) were associated with staphylococcal phages. Specifically, phage holin was given the highest classification weight and had a higher presence in strains from HE. Toxin genes, representing 10% of the feature genes, also emerged as keys in the differentiation, with many originating from prophages. Interestingly, 20% of the genes mainly found in strains derived from AD patients were hypothetical, suggesting a need to further explore *S. aureus* functions in AD, even though *S. aureus* is a widely studied model organism.

### Prophage genes significantly contribute to the genetic differences between *S. aureus* strains

**Fig 3:**
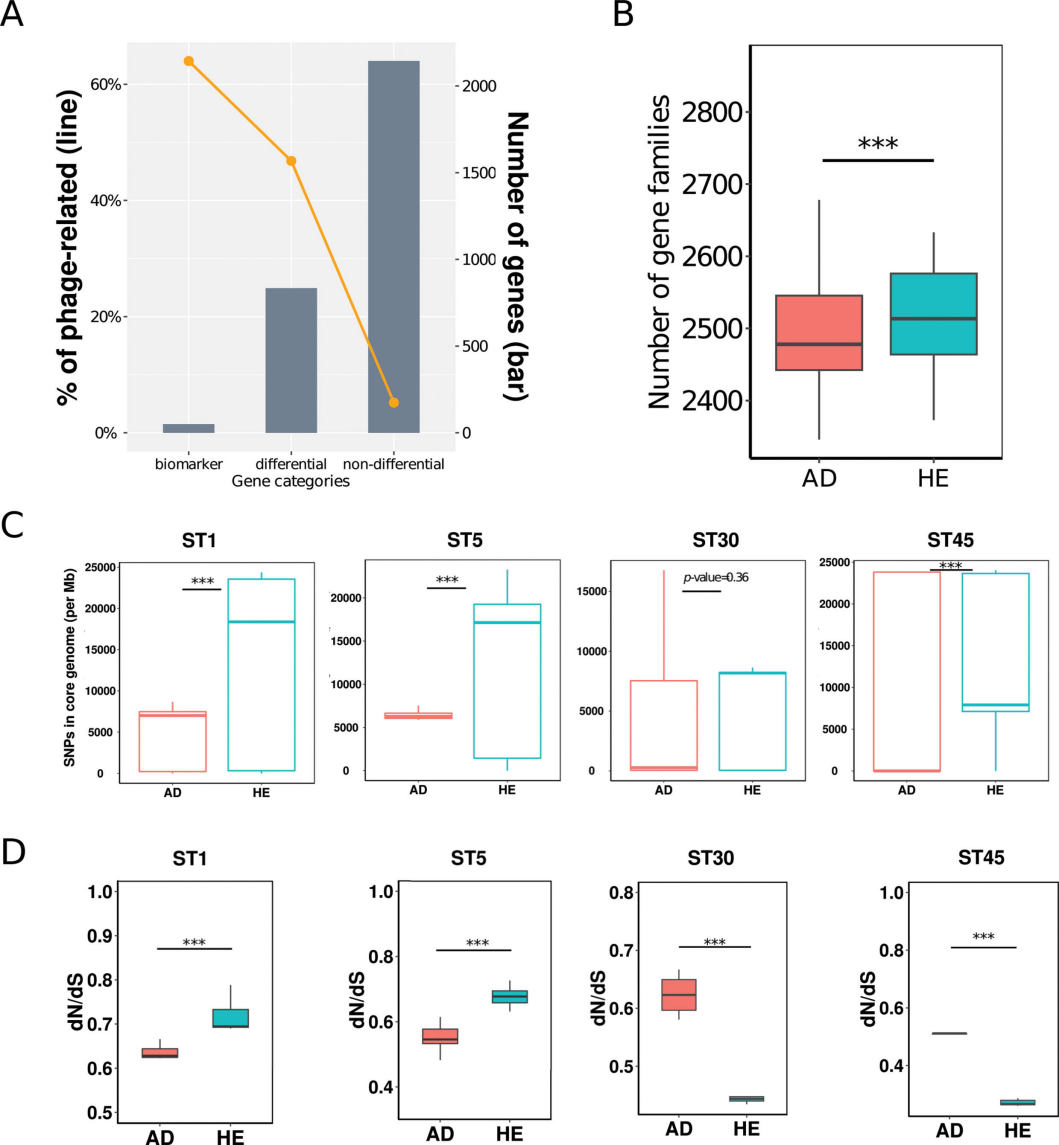
Prophages significantly contribute to the genetic differences of *S. aureus* strains. (**A**) Proportion (left) and number (right) of prophage-related genes in the marker, differential, and non-differential gene sets. Differential genes are those that contribute to the differentiation of AD and HE strains indicated by the RF classifier. Non-differential genes are the opposite. (**B**) Number of gene families of AD and HE strains. (**C**) Number of single-nucleotide polymorphisms (SNPs) calculated using SNP-dists and (**D**) ratio of dN/dS calculated using codeML, based on the core genomes of the four balanced STs (ST1, 5, 30, 45), respectively. The significance was performed by the Wilcoxon rank sum test, following the Kolmogorov-Smirnov test for the normality test of the data. *** means a *P*-value <0.001.

Having observed the significant contribution of prophages to the feature gene set, we sought to investigate how prophage genes influence the gene content of *S. aureus* genomes. We only focused on the non-rare gene families (present in >10% of strains) to enhance the generalizability and robustness of the model and reduce stochastic effects, as the RF classifier assigned an importance value to each gene family in the process, which was classified into two categories as differential (positive contribution to differentiation between AD and HE groups) and non-differential. Of these, 838 gene families were differential and 2,144 non-differential (Fig. 3A). We identified prophage genomes in most human-derived *S. aureus* strains (Table S3). By blasting the *S. aureus* genes against the predicted prophage genes, we revealed a striking shift from 5.2% phage-related genes in the non-differential gene set to an elevated 46.8% in the differential set. Besides, gene content analysis revealed a significantly higher number of gene families in the strains from HE compared to the strains from AD (Fig. 3B) despite similar genome size (Fig. S8).

### *S. aureus* micro-diversity is influenced by both health status and STs

To further explore the micro-diversity in strains from AD and HE, we assessed the number of single-nucleotide polymorphisms (SNPs) and dN/dS ratio derived from the core genome of *S. aureus* strains with matched STs present in both AD and HE groups, which could minimize the potential bias induced by asymmetrical clonal structure. A total of 45 STs were identified, of which 14 STs (244 strains) were present in both AD and HE groups (AD: 137 vs HE: 107 strains, Table S1). SNPs shed light on genetic variability, while dN/dS reveals evolutionary pressures. Typically, a dN/dS <1 suggests purifying or negative selection, with values closer to zero indicating a more intense purifying selection (21). The AD group exhibited significantly fewer SNPs and a lower dN/dS ratio than the HE group (Mann-Whitney *U* test, *P*-value  < 0.001 for both, Fig. S9), suggesting stronger purifying selection pressures in the specific skin health status the strains exposed.

To further reduce the influence of relative genetic distance, we performed a detailed examination at the ST level that highlighted four STs (ST1, 5, 30, and 45) with a relatively balanced distribution between AD and HE groups (Table S1). For SNPs, all four STs demonstrated fewer numbers in AD strains (Fig. 3C), aligning with the combined trend of the overall data set (Fig. S9). For the dN/dS analysis, however, individual analysis of the four STs revealed divergent trends; ST1 and ST5 mirrored the collective trend, while ST30 and ST45 showed opposite trends (Fig. 3D). This divergence hints at the possibility that different STs might react distinctly under their specific environmental pressures such as different skin conditions, thus both health status and strains STs might influence *S. aureus* micro-diversity.

### Prophages from *S. aureus* strains of AD patients and HE differ in gene content and functional implications

**Fig 4:**
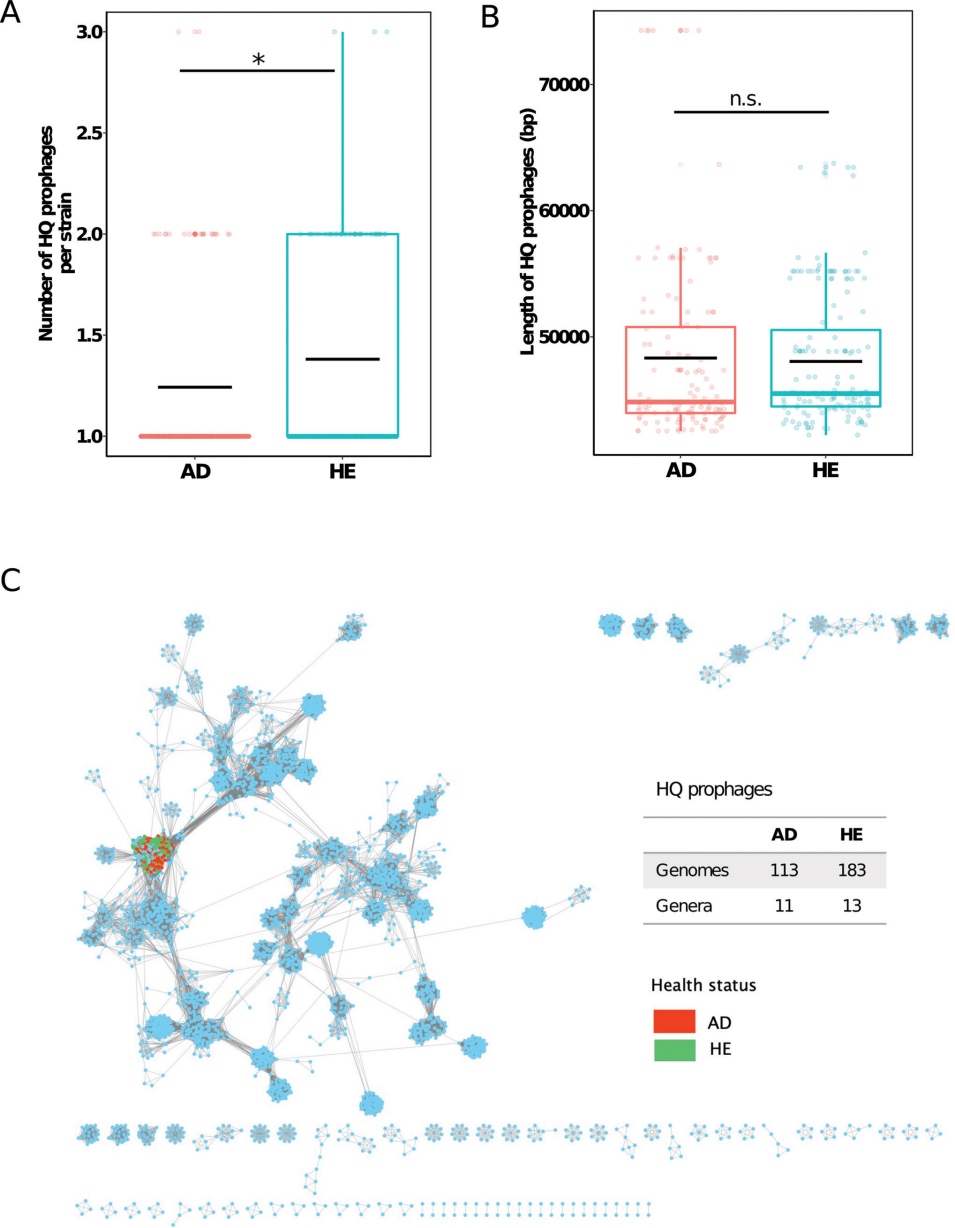
High-quality prophages in AD and HE groups display comparable size and taxonomy but differ in number. (**A**) Number of high-quality prophage sequences per strain, based on the combined prediction results of PhiSpy, VIBRANT, and Phigaro. Black crossbar in the box indicates the average number. (**B**) Length of high-quality prophage sequences predicted in AD and HE groups. Black crossbar in the box indicates the average number. The significance of the difference was performed by the Wilcoxon rank sum test. * means a *P*-value <0.05. n.s. = not significant. (**C**) Clustering analysis of high-quality prophages using vConTACT2. Each circle (node) represents a prophage sequence, and connecting lines (edges) represent the similarity between sequences based on shared clusters of proteins. Sequences are clustered at the genus level. Red and green dots represent prophage sequences predicted in the *S. aureus* strains from AD and HE groups, respectively. Light blue dots represent prophage sequences from the reference database. Number of prophage sequences and genera for AD and HE strains clustered in the network is shown on the right.

Our findings highlighted the significant role of prophages in the genetic diversity of *S. aureus* strains. By profiling the prophages of *S. aureus*, combining high-quality (HQ) genomes (completeness >90%) and all predicted sequences (including remnants), we sought to uncover their prevalence and potential genetic influence, offering precise functional insights and a deeper understanding of their evolutionary and functional roles in *S. aureus*. First, we observed the omnipresence of prophage sequences in our strains, with every strain from HE and all but seven strains from AD containing predictable prophage sequences (Table S3). When focusing on HQ prophages, we identified 133 and 163 HQ prophage genomes in AD and HE strains, respectively (Table S3). A significantly higher number of HQ prophages per strain were observed in the HE group (Wilcoxon rank sum test, *P*-value <0.05, Fig. 4A). However, no major difference in HQ prophage length was observed between the two groups (Fig. 4B). Our network analysis of the HQ prophages did not reveal dissimilarities at the genus level between AD and HE strains of *S. aureus* (Fig. 4C).

**Fig 5:**
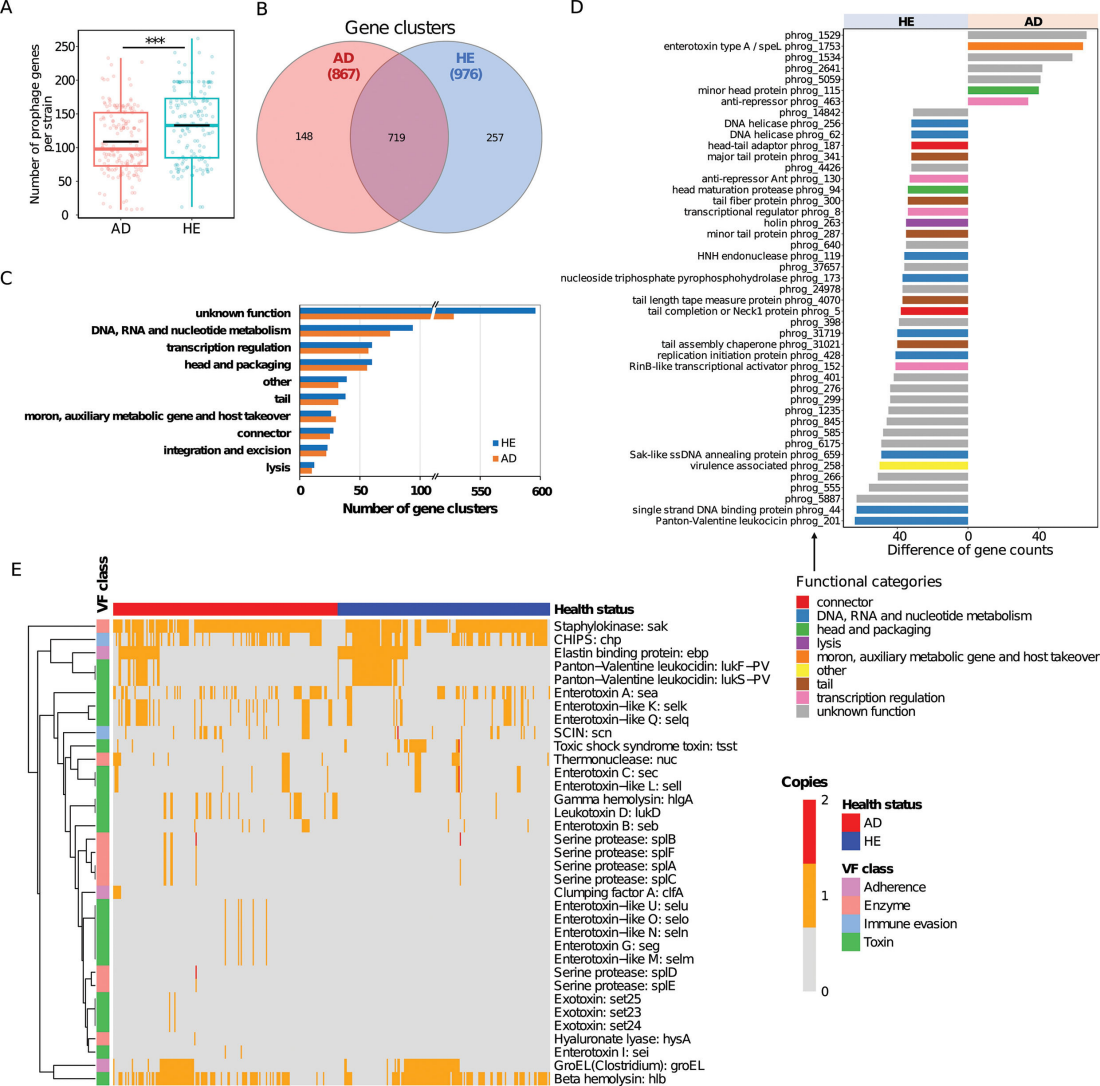
Prophage genes in AD and HE groups differ in gene content and associated functional implications based on all predicted prophage sequences. (**A**) Number of prophage gene clusters each strain possesses, assigned using MCL. Black crossbar in the box indicates the average number. * means a *P*-value <0.05. (**B**) Number of shared and unique prophage gene clusters in AD and HE groups, respectively. The number in the parenthesis represents the total number of prophage clusters identified. (**C**) Number of prophage gene clusters assigned into PHROG functional categories for AD (orange) and HE (blue) prophages. (**D**) Differential functions between AD and HE prophages (gene count difference >30), annotated with PHROG database. Bar colors show functional categories. (**E**) Heatmap of virulence factors carried in prophages of *S. aureus* strains in AD and HE groups, identified using VFanalyzer. Heatmap colors indicate the number of gene copies. Row clustering was calculated with the Euclidean method.

To fully characterize the prophage gene content as well as functional implications, we analyzed all predicted prophage sequences in more detail. Prophage gene content in HE strains exhibited significantly richer diversity (Wilcoxon rank sum test, *P*-value <0.0001, Fig. 5A), with 976 clusters identified (257 unique to the tested strains) compared to 867 clusters (148 unique) in the AD group (Fig. 5B). This result, combined with the pronounced core genome diversity of HE-derived strains (Fig. 3C and D), points to a potential co-evolution between the bacterial genome and its prophages in HE strains. Using the PHROG database for functional implications of the prophages, most functional categories were more abundant in HE-associated prophages than in AD (Fig. 5C), except for the moron, auxiliary metabolic gene, and host takeover that slightly predominated in the prophages from AD. This underscores that strains from HE might have encountered more diverse environmental challenges or possess a longer evolutionary lineage, which led to the assimilation of versatile functional genes. Examining differential functional genes of prophages between AD and HE, we pinpointed 45 genes with a differential count greater than 30 using the PHROG database (Fig. 5D); seven genes were more prevalent in prophages from AD, while 38 were predominant in prophages from HE. Notably, the category related to DNA, RNA, and nucleotide metabolism was more abundant in HE prophages, while virulence traits such as enterotoxin type A were more concentrated in AD prophages, suggesting these prophages might amplify the virulence potential of their bacterial hosts.

**Fig 6:**
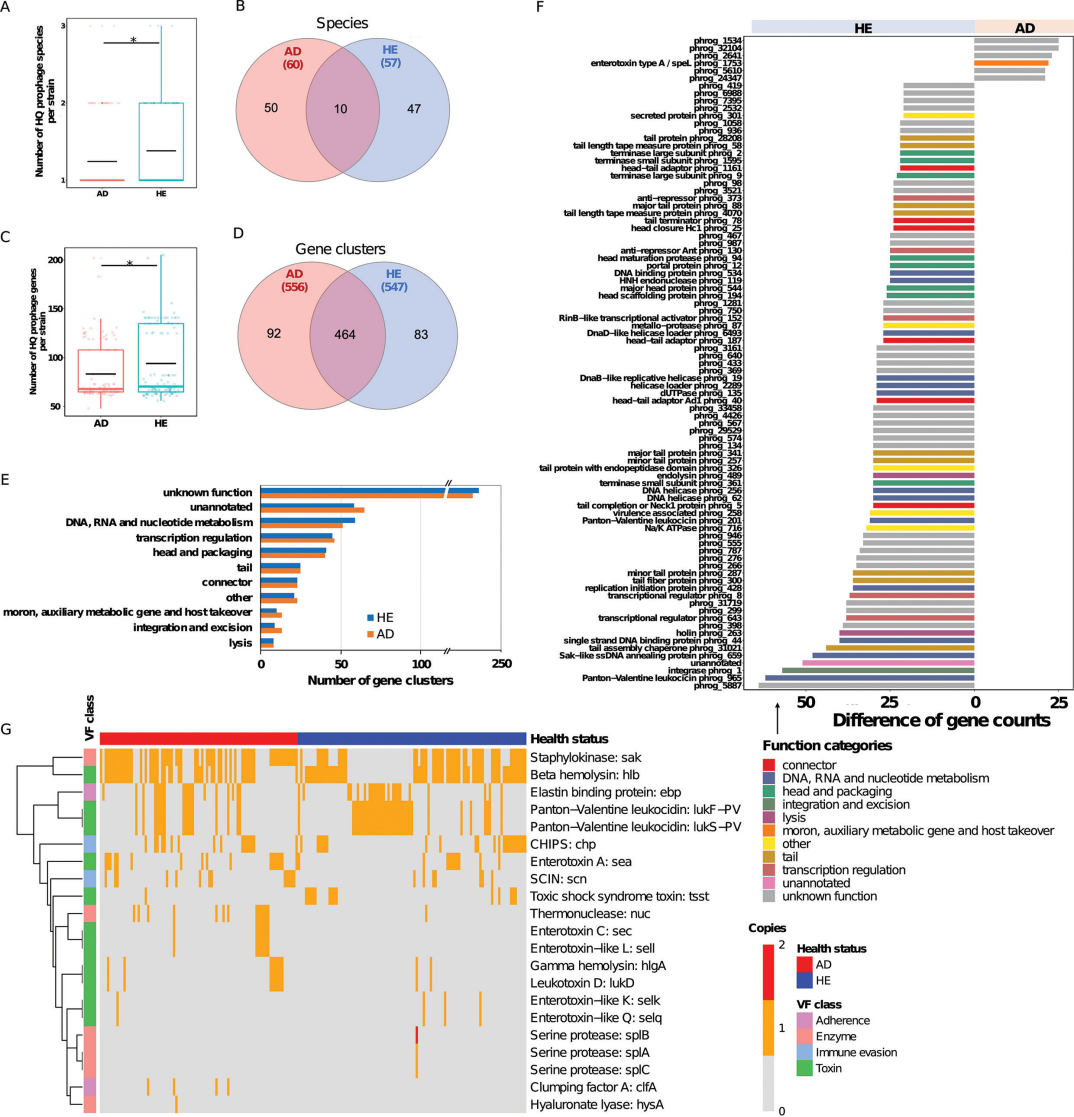
High-quality prophages in AD and HE groups differ in gene content and associated functional implications. (**A**) Number of high-quality phage species each strain harbored, identified by sequence clustering using CD-HIT with the 95% similarity cutoff. Black crossbar in the box indicates the average number. * means a *P*-value <0.05. (**B**) Number of shared and unique species for AD and HE prophages, respectively. The number in the parenthesis represents the total number of prophage species identified. (**C**) Number of high-quality prophage gene clusters each strain possesses, assigned using MCL. Black crossbar in the box indicates the average number. * means a *P*-value <0.05. (**D**) Number of shared and unique prophage gene clusters in AD and HE groups, respectively. The number in the parenthesis represents the total number of prophage clusters identified. (**E**) Number of prophage gene clusters assigned into PHROG functional categories for AD (orange) and HE (blue) prophages. (**F**) Differential functions between AD and HE prophages (gene count difference >20), annotated with PHROG database. Bar colors show functional categories. (**G**) Heatmap of virulence factors carried in prophages of *S. aureus* strains in AD and HE groups, identified using VFanalyzer. Heatmap colors indicate the number of gene copies. Row clustering was calculated with the Euclidean method.

We further profiled HQ prophage genomes in *S. aureus*, analyzing gene content, species, and functional implications for a more biologically meaningful perspective. We identified 107 prophage species using the 95% similarity cutoff. The number of prophage species per strain was significantly higher in HE than in AD group (Fig. 6A), with AD-associated strains in total housing 60 species (50 unique to the tested strains). HE-associated strains possessed 57 species, of which 47 were unique (Fig. 6B). Additionally, HQ prophages in the HE group demonstrated a significantly richer diversity of gene clusters per strain, which is consistent with the result of all predicted sequences, while a total of 556 clusters (92 unique) were identified in the AD group compared to 547 clusters (83 unique) in the HE group (Fig. 6C and D). Notably, two more known functional categories, transcription regulation as well as integration and excision, were more abundant in the HQ prophages of AD strains, besides moron, auxiliary metabolic gene, and host takeover. Differential functional gene analysis (Fig. 6F) identified enterotoxin type A was also more prevalent in HQ prophages of AD strains, while the category related to connector and DNA, RNA, and nucleotide metabolism was more abundant in HE prophages. These findings largely align with those from all prophage sequences.

### Virulence factors enriched in prophages from AD-associated *S. aureus* strains

Having observed a strong correlation between virulence factors (VFs) and prophage genes (Fig. 2 and 5D), we speculated VFs in prophages might significantly influence *S. aureus* virulence. Examination of VFs carried out in all prophage sequences indicated a higher representation of enzyme and toxin-linked VFs in AD strains, whereas adherence and immune evasion VFs were more prevalent in HE strains (Fig. S10). In HQ prophages, enzyme-related VFs were more common in the AD group, while toxin-related VFs predominated in the HE group (Fig. S11).

Analyzing the specific VF genes in all prophage sequences in more detail, over 96% of prophage-containing strains in both groups encode VFs in their prophages, underscoring the omnipresence of prophage-carrying VFs. As Fig. 5E depicted, over 70% of VFs were predominantly or exclusively associated with AD prophages, while a mere 16% were more prevalent in HE prophages. AD-associated VFs predominantly include serine protease-like enzymes (*splA - splF*), which degrade fibrinogen and plasma proteins, weakening skin barriers and enhancing inflammation. Enterotoxins (*sea - sec, seg, sei*) and enterotoxin-like enzymes (*SelKMNOPQU*), also AD dominant, cause food poisoning and provoke severe inflammatory responses. The role of exotoxins (Set23 - Set25), unique to AD prophages, might induce a substantial immune response and inflammation. Clumping factor A (*clfA*), exclusive in AD prophages, aids bacterial adherence and enhances colonization. Hyaluronate lyase (*hysA*) deteriorates the skin barrier, promoting bacterial invasion. Three other significant AD-dominant VFs are gamma hemolysin (*hlgA*) and leukotoxin D (*lukD*), both of which damage immune cells and weaken skin defense, and staphylococcal complement inhibitor (SCIN), which protects *S. aureus* from the immune system. Overall, the prominence of these VFs in AD-associated prophage sequences underscores the critical role of prophages in the pathogenicity of *S. aureus* strains in AD patients.

HE-associated prophages primarily contain VFs, such as the chemotaxis inhibitory protein (CHIPS), which blocks immune cells from infection sites. Elastin-binding protein (*ebp*) promotes bacterial adherence and tissue invasion. Panton-Valentine leukocidin (*LukF-PV* and *LukS-PV*) targets leukocytes, contributing to severe skin infections. The toxic shock syndrome toxin (*tsst*), highly abundant in HE prophages, is a potent superantigen triggering a strong immune response, potentially causing toxic shock syndrome.

The analysis of VFs encoded by HQ prophages indicates a divergence in prevalence between AD and HE strains (Fig. 6G). VFs such as staphylokinase (*sak*), thermonuclease (*nuc*), and SCIN are predominantly found in AD strains. Toxins-related genes, such as *hlgA* and *lukD,* also show higher occurrence in AD. Uniquely present in AD strains are genes such as *clfA*, *hysA*, *sec*, and *sell*. In contrast, genes, such as *ebp*, beta hemolysin (*hlb*), CHIPS, and both *lukF-PV* and *lukS-PV*, exhibit a higher presence in HE strains, echoing the trends observed across all prophage sequences. Notably, *splA - splC*, *selk*, and *selq* were unexpectedly more prevalent in HE strains based on HQ prophages, deviating from the overall prophage findings. These patterns underscore the potential role of specific genes in the pathogenesis of AD, largely aligning with the comprehensive prophage data.

## DISCUSSION

*S. aureus* has been correlated with the severity of AD conditions with abnormally high abundance on the skin of AD patients (22–24) but also colonizes the nares of healthy individuals (9). Whole-genome sequencing offered an unprecedented resolution for investigating the subtle genomic differences of *S. aureus* in clinical contexts on the level of bacterial strains. The current study aimed to investigate the genetic differences between *S. aureus* strains associated with AD and those found in HE. Utilizing RF classifiers and in-depth prophage profiling, our research revealed a set of marker genes that can accurately differentiate AD-associated strains from HE-associated strains, which further highlighted the pivotal roles of prophages in the genetic differentiation and pathogenicity of *S. aureus* in AD conditions.

While a correlation has been observed between certain *S. aureus* strains and the health status of AD patients (12, 13, 25), distinguishing *S. aureus* strains from AD and HE by comparing their whole genomes remains challenging (14). This suggests that specific genes might be responsible for their phenotypic variation, and the identification of these marker genes would be critical to reveal strain-level differentiations. Previous studies have utilized multiple typing methods to answer whether there is a relationship between certain *S. aureus* clone complexes (CC) or sequence types and AD disease but failed to identify a largely dominant clone or type for isolates from AD patients. Multi-locus sequence typing (MLST) method indicated that sequence types ST188 (19.4%) and ST1 (13.9%) were the most AD related (26), while a recent extensive analysis revealed no single ST predominates in AD on a global scale (27). Clonal complexes typing proved CC1 (20%) as the most prevalent in AD in one study (25), while it identified CC30 (33%) as the largest fraction in another recent study (13). Overall, even the most prevalent clone or type assigned to AD with these methods was less than 50%, indicating their insufficient power for differentiating AD-related *S. aureus* strains.

Here, for the first time, we have identified a concise set of marker genes that displayed high reliability in distinguishing AD-related strains with extremely high precision (Fig. S2). This represents a great advance in the differentiation of AD-related *S. aureus* strains. Our results open an exciting new avenue of study focused on the key biomarker genes to identify subtle differences between disease- and health-associated *S. aureus* strains. These biomarker genes may also serve as potential targets for therapy. Manipulating these genes could possibly shift the microbiome in a direction that is more typical of healthy individuals, thereby mitigating AD symptoms or progression.

Nevertheless, the absence of 10 feature genes in certain strains within the real-world data set challenges the accurate prediction of HE strains, underscoring the specificity and variability inherent in *S. aureus* strains. In light of this, researchers utilizing our model for studies on *S. aureus*, especially in the context of AD, may consider tailoring the feature gene set, which could involve a meticulous re-evaluation of the gene set to include additional markers that are more representative of the diversity within HE strains. Furthermore, we see the potential for the principles and methodologies of our model to be adapted and applied to other bacterial species or strains, enabling the development of custom marker gene sets. This adaptability not only enhances the model’s applicability across various microbial research scenarios but also contributes a versatile framework for strain differentiation and genomic analysis.

### Influence of clonal structure on *S. aureus* micro-diversity

Previous studies showed no significant difference in SNPs between strains from AD and HE cohorts (25). However, our micro-diversity analysis of *S. aureus* strains with matched STs showed significantly fewer SNPs and lower dN/dS ratio in AD strains than those from HE, which is in line with the reduced gene content diversity of AD strains (Fig. 3B) and may demonstrate evidence of purifying selection and adaptation compared to controls. These differences in evolutionary dynamics between AD and HE groups are crucial for understanding *S. aureus* genetics in different host settings.

*S. aureus* is known to have diverse clonal complexes or STs that show differential prevalence between AD patients and healthy individuals (28, 29). Does the collective pattern at the cohort level hold true for single STs, or does it differ among different STs? Interestingly, when examining specific STs, we observed that SNPs demonstrate a consistent pattern across different STs with all four studied STs (ST1, ST5, ST30, and ST45) exhibiting fewer SNPs in the AD group, suggesting that health status rather than STs plays a key role in the discrepancy. For accurate dN/dS comparisons between closely related strains and species, it is essential to consider the time elapsed since their divergence (30). To minimize the effect of divergence time, we also conducted intra-ST comparisons. The dN/dS ratios for ST1 and ST5 were congruent with the collective trend, whereas ST30 and ST45 showed deviations, suggesting different STs experience varying levels of purifying selection pressure or potential bias due to the limited number of strains at the ST level, particularly for ST30 and ST45. Overall, this contrast highlights the intricate evolutionary dynamics within *S. aureus*, indicating that clonal lineages may have an impact on the dN/dS ratio but exert minimal influence on SNPs. Further verification requires analysis using a more diverse and structured set of strains.

### Different prophages infect strains from AD and HE

Phages can control bacterial population dynamics via different strategies (31), which exhibit profound influences on the abundance and diversity of bacteria (32). Phages are estimated to be 20%–30% more abundant than bacteria, and they are responsible for an astounding 10^24^ infections of bacteria per second (33). Prophages greatly affect the bacterial genome architecture as well as anchor points for genome rearrangements and disrupting genes. Especially, prophage indicates past interactions of the host bacteria with phages and, in turn, their characteristic evolutional traits (34). Our study revealed distinct prophage species and richer gene contents in *S. aureus* strains from HE compared to AD, suggesting a more substantial genetic affection by prophages for the HE group over their evolutionary history. The potential co-evolution grants strains from HE an extended palette of responses to external pressures, providing them with heightened adaptability. Such a broadened adaptability can underpin survival in diverse environmental conditions or resilience against an array of antibiotics or treatments. Contrarily, strains from AD seem to be under selective pressures favoring particular *S. aureus* strains that thrive on specific AD conditions, resulting in limited gene content. The differences in gene contents (Fig. 3 and 5) were highlighted in the marker genes. Most notably, phage holin was identified as the most discriminative factor for differentiation and significantly more contributive than other genes, with a high enrichment in strains from HE. Holins, encoded in bacteriophage genomes, determine the end of phage’s replication cycle (35, 36). They create pores in bacterial cytoplasmic membranes to facilitate the release of endolysins that break down the cell wall and trigger bacterial cell death, thereby promoting virion release (37). Enrichment of phage holins in strains from HE could signify the loss of these genes in AD strains, reflecting evolutionary pressure to retain specific prophage-encoded virulence genes while shedding others. Alternatively, it may indicate increased phage activity, potentially restraining *S. aureus* proliferation and contributing to lower absolute numbers in HE.

### Functional implications of different prophages for *S. aureus* strains from AD and HE

Prophages, as integrated bacteriophage sequences within bacterial genomes, can also significantly shape bacterial physiological traits and introduce new functions by HGT (38, 39). In the context of *S. aureus*, prophages can carry a large proportion of various virulence factors, or metabolic pathways, thereby affecting the adaptability and pathogenicity of *S. aureus* (40). While prophages from HE demonstrate significantly more diverse gene content, across both data sets of HQ prophage genomes and all prophage sequences (including remnants), we found a stronger association between certain VFs and AD-associated prophages (Fig. 5 and 6), such as *clfA*, *nuc*, and several toxins (*sea, sec, sell, hlgA, lukD*) across both data sets, indicating a selective advantage with the enrichment of these VFs in AD strains. ClfA is a fibrinogen-binding protein that facilitates bacterial adherence to host tissues and plays a critical role in the pathogenesis of infections by promoting cellular aggregation (41). Nuc is a heat-stable enzyme that aids in immune evasion or biofilm formation (42, 43). Enterotoxins and enterotoxin-like enzymes (*sea*, *sec*, and *sell*) can cause food poisoning and have superantigenic properties leading to a massive inflammatory response (44). HlgA is a two-component toxin that lyses a wide range of host cells, contributing to tissue damage (45), while LukD is a pore-forming cytotoxin targeting leukocytes, disrupting the immune response (46).

The all-prophage sequences data set shows a greater diversity of VFs, especially serine proteases, enterotoxin-like factors, and exotoxins that were almost uniquely present in AD-associated prophages, suggesting that remnants contribute to virulence variability and potentially to *S. aureus* adaptability. The depth of bacterial penetration is influenced by virulence factors, specifically serine proteases, which could cause deeper inflammation that is harder to treat (47, 48). The specific roles of exotoxins have not been extensively described in the literature (49), but many SETs act as superantigens activating a significant proportion of the T-cell population and massive cytokine release and inflammatory reactions (49). However, it is not clear whether the VFs carried in prophage remnants contribute to bacterial physiology or if the prophages themselves are inducible. Surprisingly, we also found that several VFs (CHIPs, *ebp*, *lukFS-PV*, and *tsst*), previously linked with AD, were more abundant in prophages from HE across both data sets. These disparities remain to be further investigated.

The increased diversity of VFs in the all-prophage data set indicates that HGT via prophage sequences could be a significant driver of *S. aureus* evolution, with implications for infection control and therapeutic strategies in AD patients. Besides, several VFs that are traditionally found in *S. aureus* chromosomal locations or pathogenicity island (SaPI) are found in the HQ prophage genomes in our analysis, such as *ebp*, nuc, and *hlb*. The identification of these VFs within prophage regions may suggest a broader distribution of these genes or potential HGT events that warrant further investigation. For example, *ebp* and β-hemolysin have been recently detected in prophages (50). It is also reported that phages can mobilize a variety of superantigen-encoding SaPI that harbor a variety of VFs, such as TSST-1 and enterotoxin B (51), which could increase the frequency of not only intra-strain and inter-strain exchange but also potential phage-SaPI exchange of VF-coding genes. This observation underscores the complexity and dynamic nature of the *S. aureus* genome, where gene mobility and HGT might blur the traditional boundaries of VF gene location. Future work will aim to dissect these complexities to refine our understanding of the genetic landscape and its implications for the pathogenicity of *S. aureus*.

### Conclusion

Given the intricate relationship between *S. aureus* strains and AD, our investigation unveils the profound influence of prophages in shaping the genomic landscape and virulence profiles of strains associated with AD. The identified marker genes, especially those associated with prophages, exhibit remarkable discriminative power in distinguishing AD-related strains from HE-related isolates. Furthermore, the enrichment of certain VFs within AD-associated prophages illustrates the significant impact of these prophages on the heightened pathogenicity of *S. aureus* in AD conditions. Conversely, the unique genes and functions in HE prophages shed light on their adaptive evolution, possibly driven by diverse environmental challenges. These findings not only elucidate the co-evolutionary dynamics between *S. aureus* and its prophages but also pave the way for targeted therapeutic interventions for AD. Future research should delve deeper into the functional roles of the identified biomarker genes and evaluate the potential of prophage-targeted therapies to modulate *S. aureus* virulence and host-pathogen interactions in AD.

## MATERIALS AND METHODS

### Genome collection of *S. aureus* strains and preprocessing

A total of 348 genomes of *S. aureus* strains from AD and HE were collected, including genomes from strains obtained from public databases (*n* = 300) and genomes from strains isolated and sequenced in our lab (*n* = 48). First, 150 *S*. *aureus* genomes from AD patients were downloaded (3 March 2022) as assemblies from the NCBI BioSample database with keywords “atopic dermatitis and *Staphylococcus aureus.*” For HE, as the BioSample database did not contain enough qualified genome assemblies, 150 *S*. *aureus* whole-genome sequencing raw read samples were downloaded (3 March 2022) from the SRA database with keywords “*Staphylococcus aureus* and healthy skin and *Homo sapiens*” and manually assembled using Spades v3.13.0 with parameters “--careful -k 55,77,99,127 --cov-cutoff auto” (52), following the download of the raw read samples using Prefetch and splitting of forward and reverse reads using Fasterq-dump included in the SRA Toolkit 3.0.0 (53). Detailed metadata of these 300 strains can be found in Table S1.

In addition, we isolated 48 *S*. *aureus* strains, including 33 strains from AD and 15 from HE in a cohort study established at the Klinikum Augsburg in Germany, which was used as the real-world test data set to further verify the model performance. The isolation and sequencing process have been described by Wang et al. (54). The study was approved by the ethics committee of the Technical University of Munich (112/16 S and 187/17 S).

The qualities of all 348 genomes were evaluated using CheckM v1.1.3 (55). Default parameters were used for all tools described below unless otherwise specified.

### Orthologous gene clustering

Open reading frames were predicted using Prodigal v2.6.3 (56). Genes from all *S. aureus* genomes were compared against each other using BLASTP version 2.12.0+ with commands -evalue 1e-5 (57). The BLAST results were then filtered to a percent identity of 70% and query coverage of 75% (58). Finally, orthologous gene clustering was performed using MCL version 14–137 with an inflation value of 2 (59).

### Random forest classifier

We used the R package *random forest* version 4.7–1.1 (60) and followed the RF tutorial from Microbiome Helper v1.0 (61) to build an RF classifier, aiming to identify the differentiating genes based on the presence-absence table of the orthologous gene family obtained from MCL and health statuses (AD and HE). Initially, to determine the optimal training-test ratio, the public *S. aureus* strains were partitioned into training and test data sets in various partitions (from 5:5 to 9:1) across 10 iterations for each. The training data set was obtained by subsampling randomly without replacement, and the remaining strains were used as the test data set. The subsampling was repeated 10 times for cross-validation to get robust predictions. Once the optimal partition 9:1 was determined, the training (90% of strains) and test data sets (10% of strains) were used for the classifier construction and evaluation, respectively. The genomes of the 48 strains obtained in our lab were used as the real-world data set to further verify the performance of the classifier.

Gene families conserved across all strains can act as noise for model performance. Therefore, the selection of an optimal subset of predictive feature genes from the training data set is essential. Only the non-rare gene families (in over 10% of strains) that are common enough to provide meaningful associations were selected for classifier training to enhance the generalizability and robustness of the model while avoiding overfitting to rare features that might not be broadly representative or predictive. During each RF model training, gene features were ranked by model-assigned accuracy weights (feature importance). Then the classifier was further optimized using the top 50 feature genes. Its performance was evaluated on the real-world data set. Multiple metrics were used to assess model performance on both data sets, including precision, recall, *F*1-score, accuracy, and area under the curve-receiver operating characteristics (ROC). The ROC curve was obtained using R package pROC version 1.18.4 (62).

### *S. aureus* strains from non-human hosts

To investigate the predictive power of the top 50 marker genes in terms of host origins, we downloaded *S. aureus* assemblies in other hosts from the BV-BRC database (12 January 2023) (63), including 110 strains from poultries, 402 strains from pigs, and 84 strains from yaks. The top 50 genes of these strains were identified using BLASTP version 2.12.0+ (57) with the same criteria for orthologous gene clustering by blasting all genes of these strains against the representative sequences of the marker genes. The PCoA analysis was performed with R packages Vegan v2.5–6 (64) and Ade4 version 1.7–16 (65). The distance was calculated with the method Jaccard, based on the marker gene presence-absence table of *S. aureus* strains in all hosts.

### Functional annotation of the marker genes

Gene functions of *S. aureus* strains were annotated using GhostKOALA version 2.2 (66) against the “genus_prokaryotes + viruses” database (1 April 2022) to obtain KEGG functional assignments. The functions of the top 50 marker genes were assigned with KEGG functions when applicable, with further complementation by annotations from the Refseq database. The most prevalent function among the best Refseq hits was considered the potential function of the corresponding marker gene.

### Micro-evolutionary analysis of *S. aureus* strains

For each isolate of *S. aureus*, the sequence type was determined using MLST 2.19.0 (https://github.com/tseemann/mlst). The *S. aureus* strains, whose STs were found in both AD and HE groups, were defined as matched STs and retained for further analysis of micro-diversity to minimize the effect of asymmetrical sampling. The core genome alignment of *S. aureus* strains was obtained using Roary v3.13.0 (67), with the parameters “-e -n -cd 95 r -v -i 70 -iv 2.” The input file for Roary was the annotation file in gff3 format generated using Prokka v1.14.6 (68), with the parameters “--genus Staphylococcus --species aureus --cpus 30 --evalue 1e-05.” Pairwise single-nucleotide polymorphisms distance matrix for *S. aureus* strains was calculated using snp-dists v0.7.0 with default settings (https://github.com/tseemann/snp-dists).

We also assessed the strength of purifying selection on *S. aureus* strains using the ratio of non-synonymous and synonymous substitutions (dN/dS). Based on the core genome alignments of strains from AD and HE, respectively, the pairwise dN/dS ratio was calculated using maximum-likelihood approximation (codeML) within the PAML v4.10.6 package (69). Values <1 indicate purifying selection with values close to 0 indicating stronger purifying selection and higher values hint at greater genetic drift (weaker purifying selection) (21). We excluded dN/dS values where dS ≥1, as this suggests synonymous substitutions are approaching saturation.

The phylogenetic tree was constructed using CVTree version-3.0.0 (70) based on the whole genomes of the 348 *S*. *aureus* strains in this study to show the clonal structure. Three genomes from *S. argenteus*, *S. epidermitis*, and *S. schweitzeri* were used as the outgroup.

### Identification and annotation of prophages

The prophage genome sequences were identified based on the combined annotations using PhiSpy v4.2.19 (71), VIBRANT v1.2.1 (72), and Phigaro v2.3.0 (73) with default parameters. The quality assessment of prophage sequences was performed using CheckV v0.8.1 (74). The taxonomy of the HQ prophages was identified using CD-HIT V4.8.1 with commands “-c 0.95 n 10 -d 0 M 0” (75) by sequence clustering, setting a 95% similarity as the species cutoff (76). Taxonomic assignment of *S. aureus* HQ prophages was performed using vConTACT v.2.0 (77), which uses protein sequence similarity to identify and cluster prophage sequences and can capture similarity at the genus level (77). Prophage genes were identified using Prodigal v2.6.3 (56). By blasting all *S. aureus* genes against prophage genes using BLASTP version 2.12.0+ (57), phage-related genes in *S. aureus* were identified with 70% identity and 75% coverage. Prophage gene clusters were identified using the same workflow for orthologous gene clustering of *S. aureus* strains. The functional annotation of the prophages identified was carried out against the PHROG database v3 (78). To reduce the number of genes with unidentified functions, each unknown gene within a given cluster was assigned the most commonly known function for the same cluster. Virulence factors of prophages were annotated with VFanalyzer in VFDB (2022) with default settings (79).

### Statistical analysis

For the statistical evaluation, the normality of the data distribution was checked in the first step either through the Shapiro-Wilk test or the Kolmogorov-Smirnov test for observations beyond 5,000. Following this, the Welch two-sample *t*-test (for normal distribution) or the non-parametric Mann-Whitney *U* test, also known as the Wilcoxon rank sum test (for non-normal distribution), was applied to determine the significance of the noted differences. A *P*-value of less than 0.05 was treated as a marker of statistical significance.

## Data Availability

The data sets supporting the conclusions of this manuscript have been included in Supplementary Tables. The real-world data set was published by Wang et al., 2022 (54). Code for the RF classifier building is available at Github: https://github.com/zhongjiew/RF-classifier-for-AD-related-Saureus.

## AFTER EPUB

*[This article was published on 16 July 2024 without a contribution note in the byline. Equal contributions were noted in the current version, posted on 06 August 2024.]*

## SUPPLEMENTAL MATERIAL

Supplemental figures spectrum.00915-24-s0001.pdf Fig. S1 to S11.

Table S1 spectrum.00915-24-s0002.xlsx Information on human-derived *Staphylococcus aureus* strains.

Table S2 spectrum.00915-24-s0003.xlsx Metadata of animal-derived *Staphylococcus aureus*.

Table S3 spectrum.00915-24-s0004.xlsx Summary of the predicted prophages of all 348 *S. aureus* strains.

ASM does not own the copyrights to Supplemental Material that may be linked to, or accessed through, an article. The authors have granted ASM a non-exclusive, world-wide license to publish the Supplemental Material files. Please contact the corresponding author directly for reuse.
