# Supplementary material for: Distinct prophage gene profiles of Staphylococcus aureus strains from atopic dermatitis patients and healthy individuals
Source: Microbiol Spectr. 2024 Jul 16;12(8):e00915-24. doi: 10.1128/spectrum.00915-24 (PMC11302291; doi:10.1128/spectrum.00915-24)
Supplement: Supplemental figures — Fig. S1 to S11. [file spectrum.00915-24-s0001.pdf]

Distinct prophage gene profiles of *Staphylococcus aureus* strains from atopic dermatitis patients and healthy individuals

Zhongjie Wang[a], Xue Peng[b,c], Claudia Hülpüsch[d,e,f], Mohammadali Khan Mirzaei[c,g], Matthias Reiger[d,e],# Claudia Traidl-Hoffmann[d,e,f],# Li Deng[c,g],# Michael Schloter[a,h] #

[a]Research Unit for Comparative Microbiome Analysis, Helmholtz Munich, German Research Center for Environmental Health, Neuherberg, Germany
[b]Faculty of Biology, Biocenter, Ludwig Maximilian University of Munich, Munich, Germany
[c]Institute of Virology, Helmholtz Munich, German Research Centre for Environmental Health, Neuherberg, Germany
[d]Environmental Medicine, Faculty of Medicine, University of Augsburg, Augsburg, Germany
[e]Insitute of Environmental Medicine, Helmholtz Munich, German Research Center for Environmental Health, Neuherberg, Germany
[f]Christine Kühne Center for Allergy Research and Education, Davos, Switzerland
[g]Chair of Prevention of Microbial Infectious Diseases, Central Institute of Disease Prevention and School of Life Sciences, Technical University of Munich, Freising 85354, Germany
[h]Chair of Environmental Microbiology, TUM School of Life Sciences Weihenstephan, Technical University of Munich, Freising, Germany

Running title: Prophage influence on *S. aureus*

#Address correspondence to Matthias Reiger, matthias.reiger@helmholtz-munich.de; Claudia Traidl-Hoffmann, claudia.traidl-hoffmann@helmholtz-munich.de; Li Deng, li.deng@helmholtz-munich.de; Michael Schloter, michael.schloter@helmholtz-munich.de

Supplementary Figures

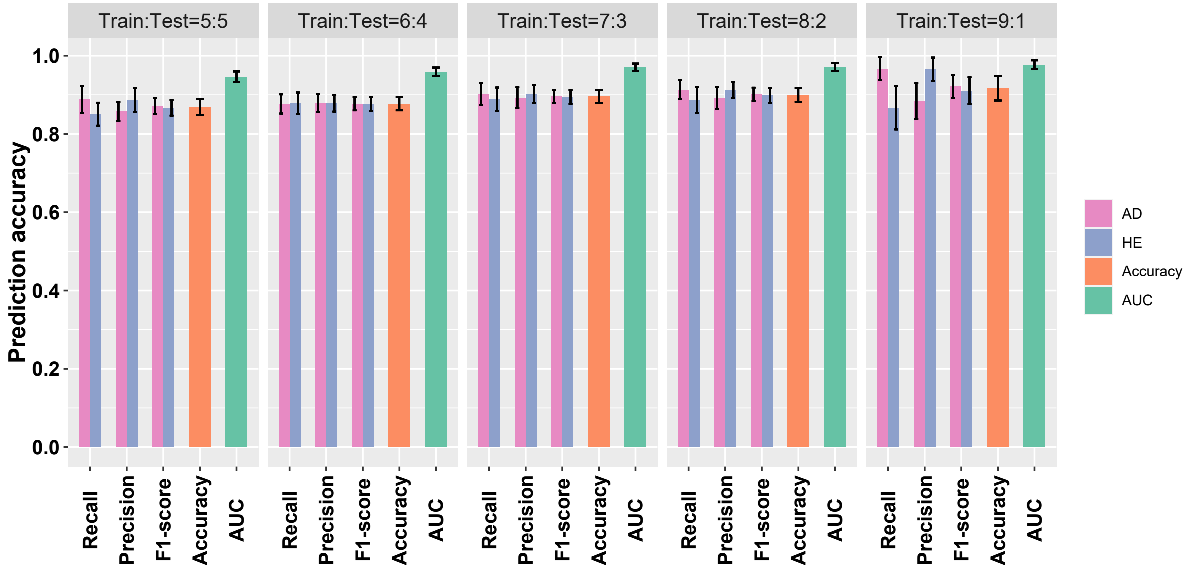

Figure S1. Determination of the train-test partition. The dataset was divided into training and test datasets in 5:5 to 9:1 proportions across 10 iterations for each. Recall, precision, F1-score, accuracy, and AUC were used to evaluate the performance of the RF models. The black bars indicate the 95% confidence intervals.

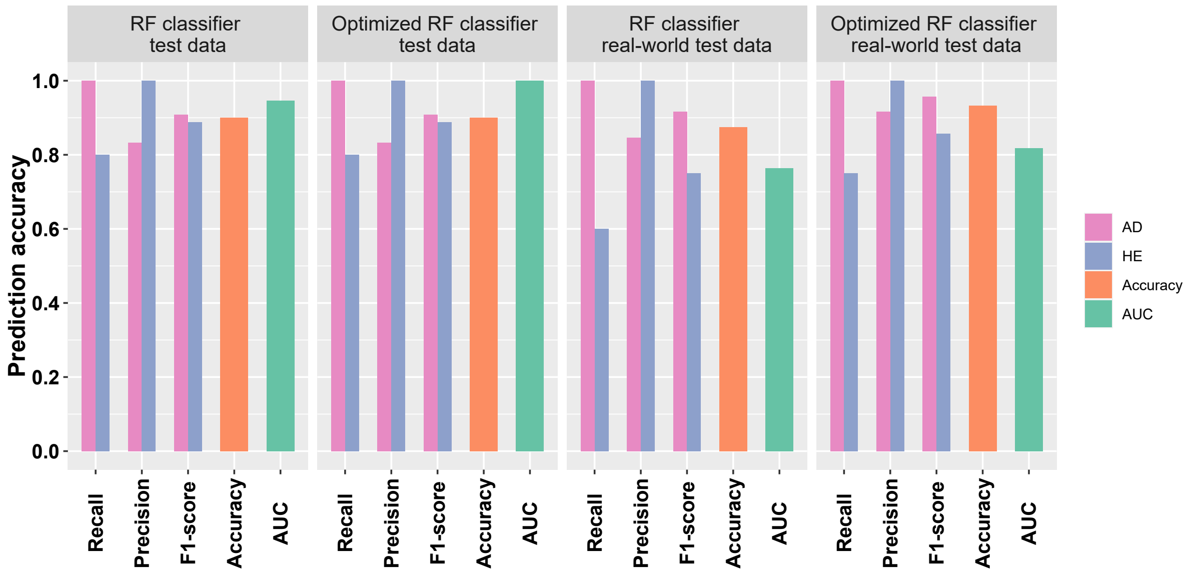

Figure S2. Performance of the RF classifier and the optimized RF classifier on both the test and the real-world test datasets. The test dataset was divided into training and test datasets in 9:1 proportion. Recall, precision, F1-score, accuracy, and AUC were used to evaluate the performance of the RF models.

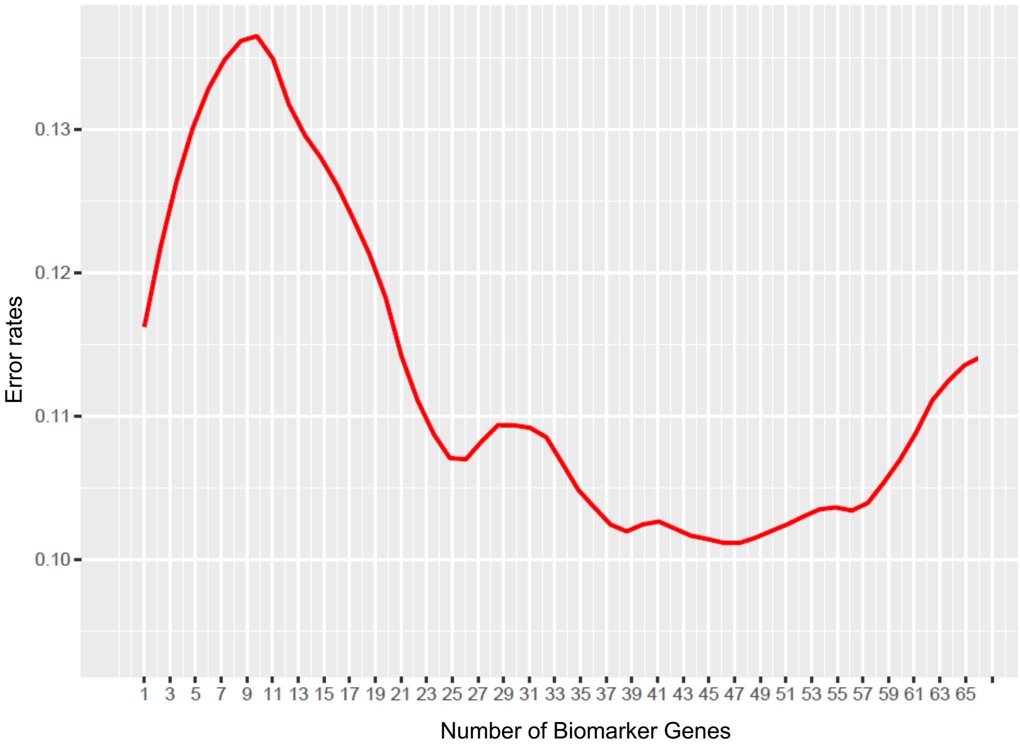

Figure S3. Selection of key marker genes via 10-fold cross-validation. The optimal prediction error rate was achieved with 50 genes. Consequently, a refined classifier was developed using the top 50 marker genes, ranked by their mean decrease in accuracy.

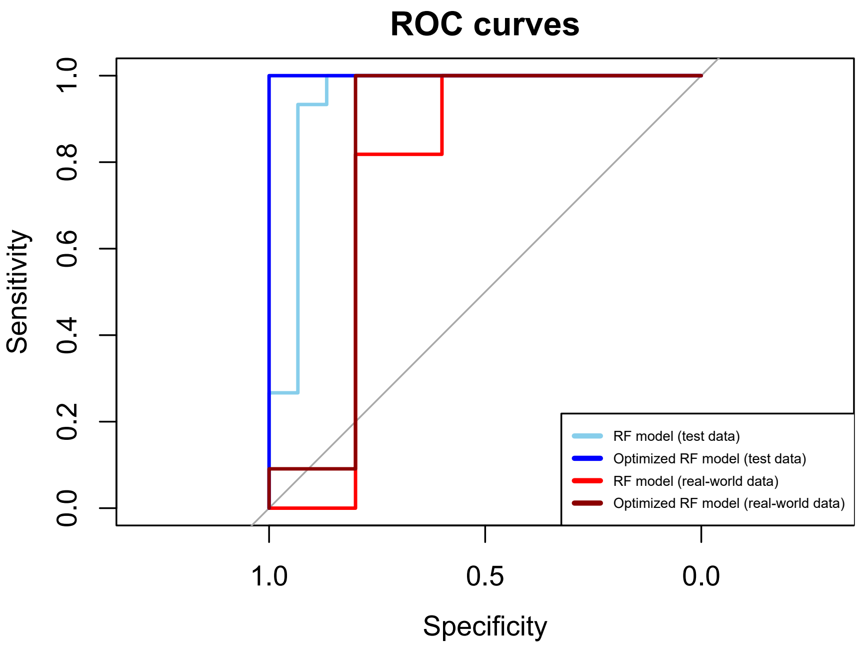

Figure S4. Receiver operating characteristic curves (ROC) for prediction of *S. aureus* strains from AD and HE groups based on the presence-absence table of gene families generated by MCL. Sensitivity is also recall. Specificity is also precision. AUC (Area under the curve) was 0.9467 and 1 for the test data when using the RF model and the optimized version, respectively, while the real-world test data achieved 0.7636 and 0.8182, respectively.

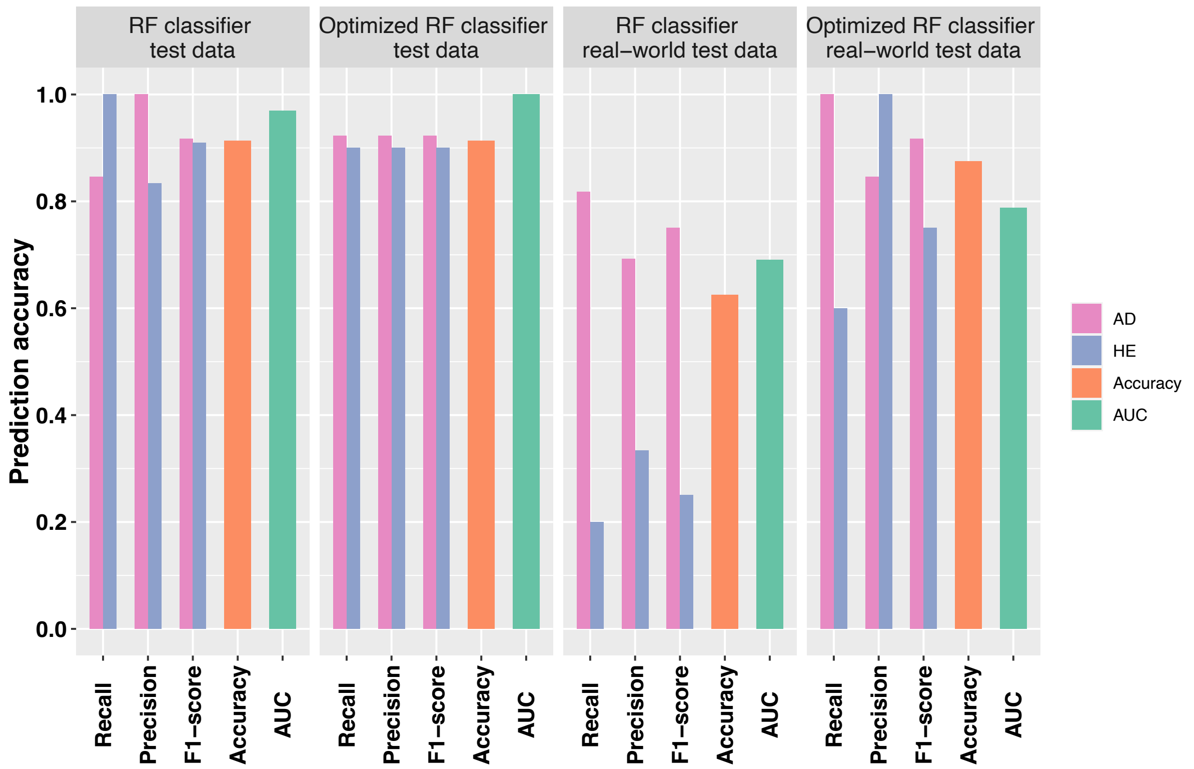

Figure S5. Performance of the RF classifier and the optimized RF classifier based on the strains with matched STs. The test dataset was divided into training and test datasets in 9:1 proportion. Recall, precision, F1-score, accuracy, and AUC were used to evaluate the performance of the RF models.

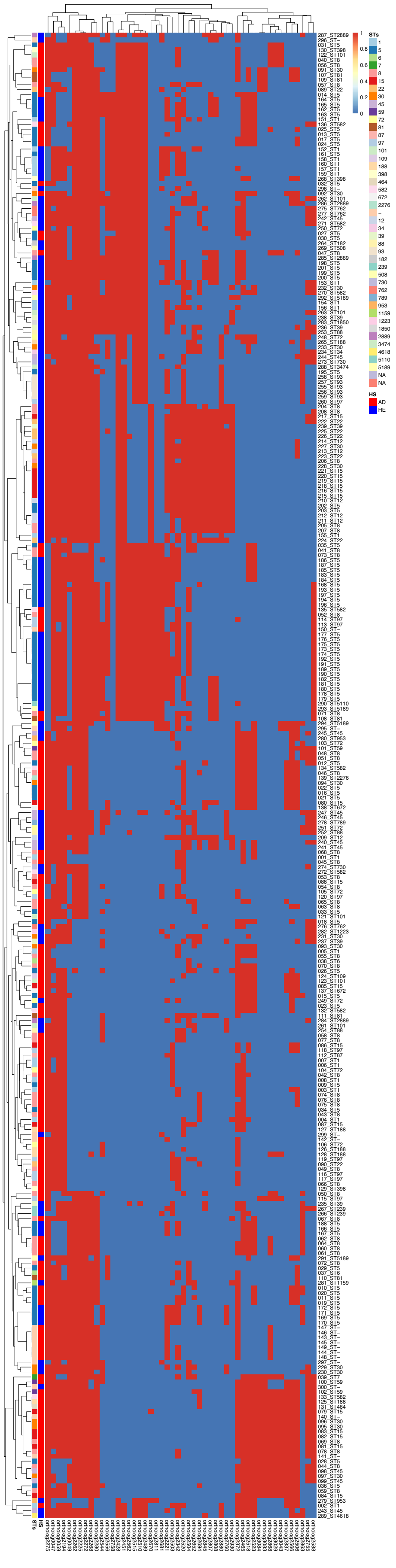

Figure S6. Heatmap of the clustering analysis of 300 publicly available strains, each labeled with its respective ST, based on the presence or absence of the 50 marker genes. STs with a high number of strains are approximately evenly distributed across clusters of strains (refer to clustering of rows), exemplified by ST1, 5, 8, 15, et al. Red indicates presence and blue indicates absence of marker genes. STs and health status of all strains (columns) were annotated beside row clusters. Labels in the right consists of two parts: genome number and specific ST assigned using MLST. Both rows and columns were clustered using the Euclidean method.

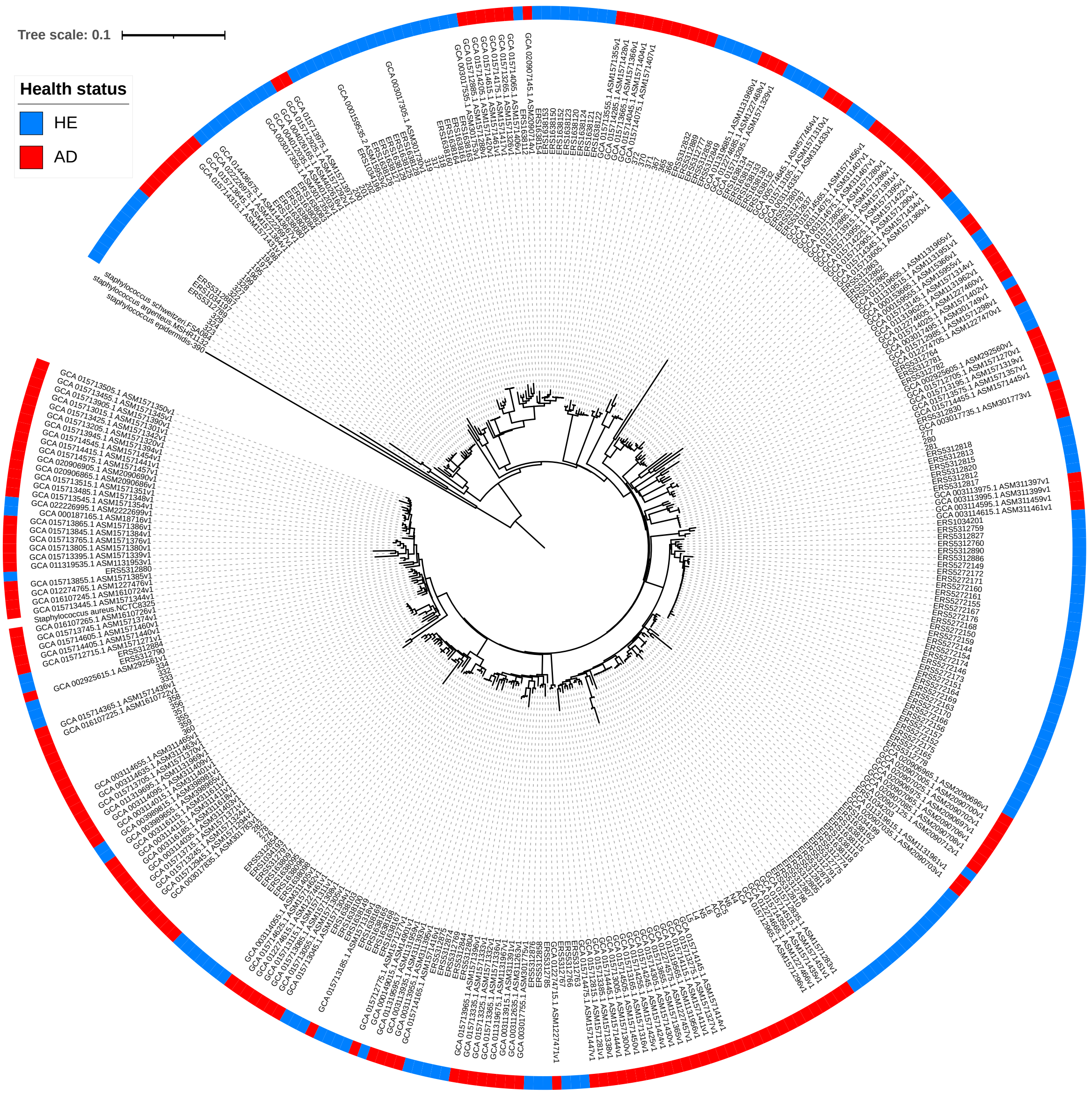

Figure S7. Phylogenetic tree based on the whole genomes of the 348 *S. aureus* strains in this study to show the clonal structure. Health status is shown for AD (red) and HE (blue) strains. Three genomes from *S. argenteus*, *S. epidermitis*, and *S. schweitzeri* were used as the outgroup.

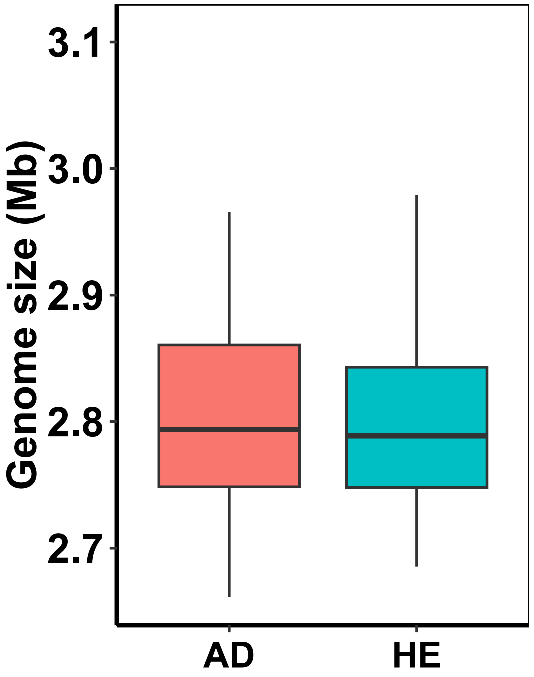

Figure S8. Genome size of *S. aureus* strains from AD and HE groups. No significant difference was detected between the two groups.

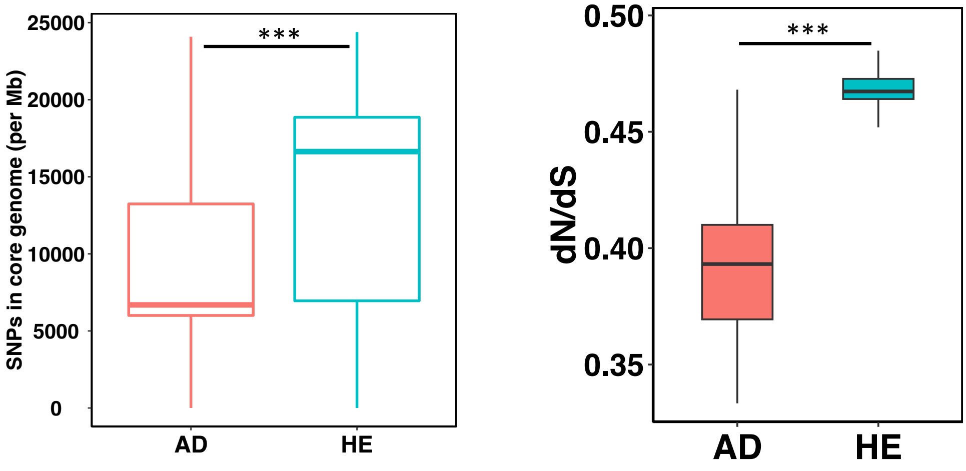

Figure S9. Number of SNPs in core genome per Mb calculated using SNP-dists, and ratio of nonsynonymous and synonymous substitutions (dN/dS) calculated using codeML based on the overall core genomes of AD and HE strains with matched STs, respectively. The significance was performed by the Wilcoxon rank sum test, following the Kolmogorov-Smirnov test for the normality test of the data. *** means a p-value < 0.001.

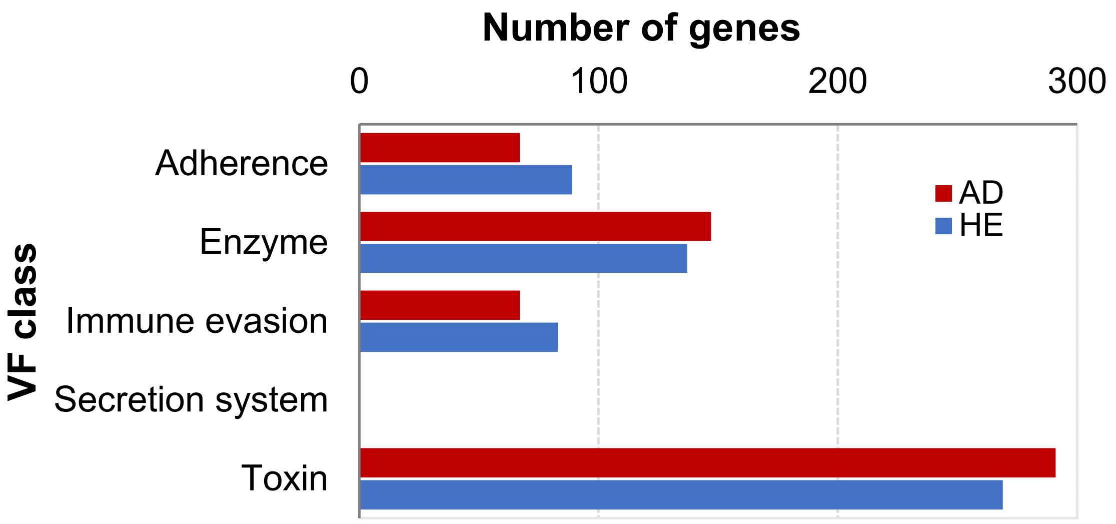

Figure S10. Number of all prophage genes assigned into each virulence factor class in AD and HE groups, respectively.

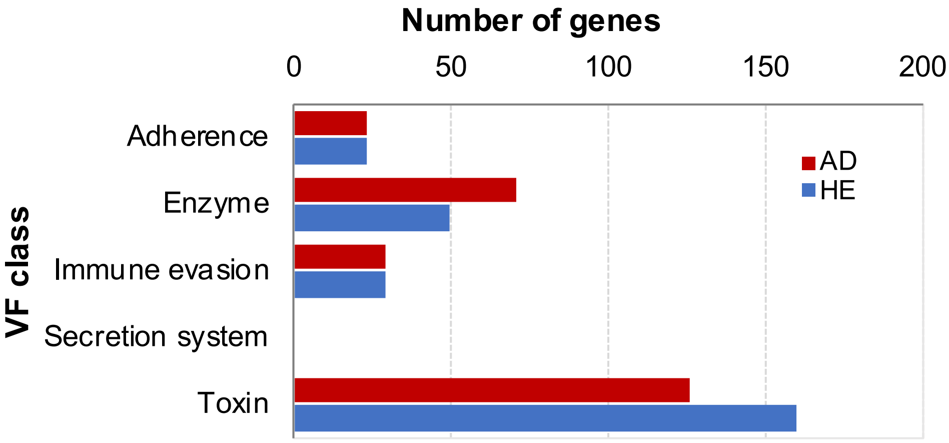

Figure S11. Number of high-quality prophage-coding genes assigned into each virulence factor class in AD and HE groups, respectively.
